# An Appetite for Destruction: Detecting Prey-Selective Binding of α-Neurotoxins in the Venom of Afro-Asian Elapids

**DOI:** 10.3390/toxins12030205

**Published:** 2020-03-23

**Authors:** Richard J. Harris, Christina N. Zdenek, David Harrich, Nathaniel Frank, Bryan G. Fry

**Affiliations:** 1 Venom Evolution Lab, School of Biological Sciences, University of Queensland, St Lucia, QLD 4072, Australia; rharris2727@googlemail.com (R.J.H.); christinazdenek@gmail.com (C.N.Z.); 2 QIMR Berghofer, Royal Brisbane Hospital, Brisbane, QLD 4029, Australia; David.Harrich@qimrberghofer.edu.au; 3 Mtoxins, 1111 Washington Ave, Oshkosh, WI 54901, USA; nate@mtoxins.com

**Keywords:** Elapidae, prey specificity, alpha-neurotoxins, neurotoxicity, venom, nicotinic acetylcholine receptor

## Abstract

Prey-selective venoms and toxins have been documented across only a few species of snakes. The lack of research in this area has been due to the absence of suitably flexible testing platforms. In order to test more species for prey specificity of their venom, we used an innovative taxonomically flexible, high-throughput biolayer interferometry approach to ascertain the relative binding of 29 α-neurotoxic venoms from African and Asian elapid representatives (26 *Naja* spp., *Aspidelaps scutatus*, *Elapsoidea boulengeri,* and four locales of *Ophiophagus hannah*) to the alpha-1 nicotinic acetylcholine receptor orthosteric (active) site for amphibian, lizard, snake, bird, and rodent targets. Our results detected prey-selective, intraspecific, and geographical differences of α-neurotoxic binding. The results also suggest that crude venom that shows prey selectivity is likely driven by the proportions of prey-specific α-neurotoxins with differential selectivity within the crude venom. Our results also suggest that since the α-neurotoxic prey targeting does not always account for the full dietary breadth of a species, other toxin classes with a different pathophysiological function likely play an equally important role in prey immobilisation of the crude venom depending on the prey type envenomated. The use of this innovative and taxonomically flexible diverse assay in functional venom testing can be key in attempting to understanding the evolution and ecology of α-neurotoxic snake venoms, as well as opening up biochemical and pharmacological avenues to explore other venom effects.

## 1. Introduction

The role of predator–prey relationship dynamics plays a key role in our understanding of how prey provide positive selection pressures for toxins and crude venom to become more prey specific. Regarding toxin diversification and evolution, the Red Queen hypothesis [1] would suggest that an arms race between predator and prey would provide a positive selection pressure for predatory venom toxins to evolve prey specificity and, reciprocally, for toxin resistance to evolve in prey and predators of venomous animals. Evidence for prey driving toxin specificity has been suggested by the discovery of taxon-specific crude venoms [2,3,4,5,6] and individual toxins [7,8,9,10,11,12,13] across the animal kingdom. Molecular evidence likewise suggests that some venom toxins are under high positive selection pressure, causing rapid diversification through mutations that alter protein structure and function [14,15,16]. These selection pressures have been reportedly due to preferential prey targets driving the selection pressures of venom potency [15,16,17,18].

Snake venoms contain a multitude of different toxin classes, all with varying pathophysiological targets [19]. In addition, snake venoms are predominantly used for predatory purposes (with the exception of some unique functions such as spitting in cobras); thus, it is likely that these co-evolutionary arms races and selective pressures are acting upon some snake venom toxins to evolve target specificity to immobilise certain prey types more efficiently.

Snake venom phospholipase A_2_s (PLA_2_s) have a wide range of pharmacological effects including anticoagulant, cytotoxic, myotoxic, and neurotoxic. Some PLA_2_s are suggested to potentially exhibit species-specific binding due to their high affinity toward specific proteins/glycoproteins rather than lipid domains on their target cells [17,20]. Presynaptically acting PLA_2_s such as β-bungarotoxin, crotoxin, and taipoxin all displayed radically different potencies between rat/mouse phrenic nerve–diaphragm (RPND/MPND) compared to chick biventer cervicis muscle (CBCM) preparation functional tests [17,21,22,23] suggesting prey-specific effects.

Other snake venom toxin types have displayed prey specificity such as the neurotoxic three-finger toxins (3FTxs). Denmotoxin and iriditoxin are 3FTxs isolated from the colubrids *Boiga dendrophila* and *Boiga irregularis*, respectively [11,12]. These toxins have shown extreme prey selectivity, with denmotoxin being 100-fold more potent toward chick muscle than that of mice [11], whilst iriditoxin showed lethality toward birds and lizards but not mice [12]. A 3FTx from the green vine snake, *Oxybelis fulgidus* named fulgimotoxin showed potency toward lizard prey but had no effect on mice [9], suggesting prey-specific effects. Two 3FTxs, α-elapitoxin-Pc1 and α-elapitoxin-Ppr1 from *Pseudechis colletti* and *Pseudechis porphyriacus,* respectively, elicit inhibition of indirect twitches on CBCM but did not have any significant effect on RPND [24], suggesting specificity toward chicken but not toward rodents. Conversely, alpha-cobratoxin, a 3FTx isolated from *Naja kaouthia,* showed no difference in lethality (LD_50_) between lizard and mouse models [25], suggesting that not all 3FTxs induce prey-specific effects. However, other prey models were not tested against alpha-cobratoxin; thus prey-specificity cannot be ruled out until investigated further.

The 3FTxs are a ubiquitous, non-enzymatic toxin class found within venoms of Elapidae [26,27] and have recently been discovered to be a major component in some Colubridae and other advanced snake venoms [9,11,12,28]. The basal action of 3FTx is to cause flaccid paralysis via blocking of the orthosteric site (acetylcholine binding region) of postsynaptic nicotinic acetylcholine receptors (nAChRs), specifically the muscle-type α-1 nAChR subunit at the neuromuscular junction [26,27,29,30]. Toxins that target these receptors are widely known as α-neurotoxins [26]. Since 3FTxs are small (60–75 amino acids), under positive diversifying selection [14], and some have been shown to display prey specificity [5,11,12,28,31], they are an ideal model to further test for prey-specific binding of toxins, which will also further our understanding of these toxins not only evolutionarily but also biochemically and pharmacologically.

In order to help our understanding of prey-specific binding of toxins and how these shape overall venom evolution, this study utilised a validated biolayer interferometry (BLI) assay [32] to test a large diversity of 3FTx-rich venoms from 29 African and Asian elapids (26 *Naja* spp., *Aspidelaps scutatus*, *Elapsoidea boulengeri*, and four locales of *Ophiophagus hannah*) and a 3FTx representative (alpha-cobratoxin) against the alpha-1 nAChR orthosteric sites from some of the major groups of potential prey types: amphibian, lizard, snake, bird, and rodent. Our results provide evidence that prey-specific binding does occur within some of the species tested at both the toxin and crude-venom level. However, it is likely that the overall proportions of prey-specific toxins within the venom play a greater role in crude-venom specificity and that diet range may moderate the overall proportions of prey-specific toxins.

## 2. Results and Discussion

**Figure 1:**
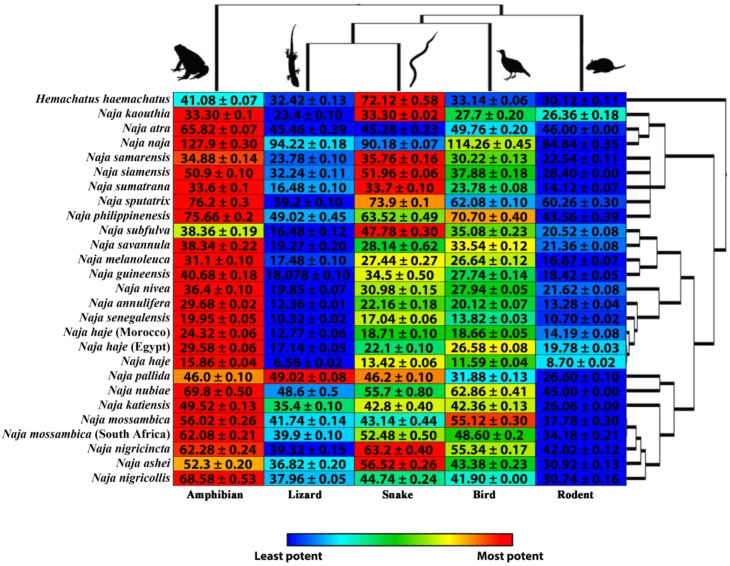
A heat–map comparison of African and Asian *Naja* species venoms, including closely related *Hemachatus haemachatus*, across all peptides. The heat map is based on the relative potency (AUC—area under the curve), derived from K_a_ (binding rate) curves in triplicate. Heat-map colours scale from red representing the highest AUC value to blue representing the lowest AUC value per species. Values are AUC ± SEM, derived from K_a_ (binding rate) curves in triplicate. *Naja* phylogeny was taken from timetree.org, which is based upon phylogenetic studies of cobras [39,40,41]. Animal images are public domain CC0 1.0 via phylopic.org.

Our data indicate that the neurotoxic nAChR binding across the majority of *Naja* tested showed some selectivity toward the amphibian mimotope (Figure 1) with 70% of *Naja* species binding more selectively to the amphibian, and this target being a close second highest in all five other *Naja* venoms that did not have amphibian as their highest binding (Figure 1). Venoms from species such as *N. kaouthia* and *Naja sumatrana* exhibited an equal highest binding for both amphibian and snake. Further, five *Naja* species (*N. ashei, N nigricincta, N. samarensis, N. siamensis,* and *N. subfulva*) exhibited their greatest neurotoxic binding toward snake mimotope but with amphibian also strongly bound.

Cobras are known to be prey generalists feeding on a range of different taxa [33,34,35,36,37]. Amphibians, reptiles (particularly snakes), and mammals are common prey types of most *Naja* species [33,34,36,37,38]. This broad diet across *Naja* species highlights their ecological plasticity and in part might explain their successful diversification across Africa and Asia. Although all *Naja* crude venoms showed some degree of binding selectivity toward a specific mimotope, for some species there were no enormous disparities between the area under the curve (AUC) values, which may be associated with the generalist nature of the *Naja* diet, thus being under a selection pressure to immobilise many different prey types efficiently. These data are interesting in that we are able to detect distinct venom binding differences between closely related species both in binding and mimotope receptor selectivity.

**Figure 2:**
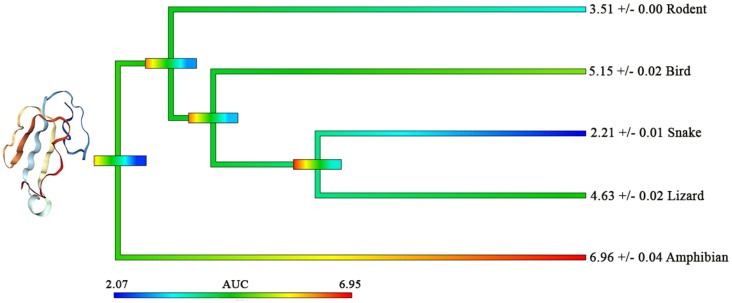
Heat–mapping of area under the curve (AUC) values for binding affinity of the α-1 nicotinic acetylcholine receptor (nAChR) orthosteric site to α-cobratoxin (obtained from *Naja kaouthia*) across various vertebrate taxa. The heat–mapping scale is coloured so lower AUC values (lower potency) are cooler, while higher AUC values (higher potency) are coloured warmer. Phylogenetic tree node bars indicate error ranges. All values are *n* = 3 mean ± SEM, derived from K_a_ (binding rate) curves. Image of α-cobratoxin (α-CTx) [P01391] taken from RCSB Protein Data Base [45], adapted from previous research [46].

Although the alpha-cobratoxin data showed some significant binding differences between the mimotopes, the AUC values do not have as much of a disparity compared to the AUC difference of the crude venom (Figure 1 and Figure 2). Thus, it is likely there is little difference in neurotoxic potency of the alpha-cobratoxin between the different prey targets. By comparing both the crude venom (Figure 1) and alpha-cobratoxin (Figure 2) data, we postulate that the species whose crude venom showed a higher selective binding to one particular mimotope would have a higher proportion of selective 3FTx isoforms toward that mimotope within their venom compared to other 3FTx isoform proportions. For example, the lowest binding of alpha-cobratoxin (Figure 2) was toward the snake mimotope, despite the crude venom targeting of *N. kaouthia* having its joint highest binding toward snake (Figure 1). Thus, a higher relative proportion of snake-specific 3FTx isoforms within the crude venom would affect the overall neurotoxic selectivity toward snake receptors more so than other prey receptors. Simply put, the expression levels/proportions of certain 3FTx isoforms are what mediate the selectivity of snake crude venoms and, thus, are what cause differential lethality or susceptibility of crude venoms between certain prey types. Future research should aim to compare crude venom vs. isolated individual toxin mimotope binding in attempting to test this idea. This is particularly relevant as the evolutionary selection pressures would act on the overall venom action, not that of an individual toxin.

Previous research testing lethality (LD_50_) of an alpha-cobratoxin variant showed no difference between mouse and lizard subjects [25]. Our data may corroborate this research since the binding values between rodent and lizard mimotope (Figure 2), with the potency against lizard only 30% above that of mouse. However, the potency against amphibian was 50% greater than that of lizard and 98% greater than that of rat. In contrast, the crude venom had amphibian binding 42% stronger than that to lizard and 26% stronger than that to rodent. Thus, individual toxins may differ from the overall selectivity of the crude venom.

Further, these results indicate that the selectivity of a particular toxin class may vary from that of the overall diet, thus suggesting that some taxa are more vulnerable to a particular toxin type and thus this component of the venom may be used for a particular function. Consistent with this, the data on cobra crude venom and alpha-cobratoxin binding (Figure 1 and Figure 2) highlight some significant preference to specific mimotopes that correlate with a major prey items but do not account for the full range of known prey items of *Naja* species [33,34,35,36,37]. Therefore, other toxins with different functions within the venom (e.g., coagulotoxic and myotoxic PLA_2_s) [42,43] may play an equally important role in biochemically immobilising different taxa types. Thus, diet breadth will most likely moderate prey specificity of crude venom to some degree, as suggested by other research [44], and the greater complexity of generalist venoms is consistent with different biochemical parts being selected for action upon particular taxon.

**Figure 3:**
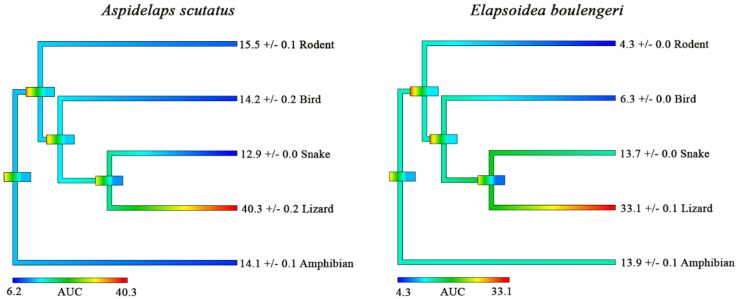
Heat–mapping of area under the curve (AUC) values for binding affinity of the α-1 nAChR orthosteric site of various vertebrates to venoms of *Aspidelaps scutatus* and *Elapsoidea boulengeri.* Phylogenetic tree colouring is represented with lower AUC values (lower potency) coloured cooler, while higher AUC values (higher potency) are coloured warmer. Phylogenetic tree node bars indicate error ranges. All values are *n* = 3 mean ± SEM, derived from K_a_ (binding rate) curves.

Our results indicate that the crude venom of both *A. scutatus* and *E. boulengeri* show strong selective binding toward the lizard alpha-1 nAChR (Figure 3). What is interesting is the large disparity between AUC values of the lizard mimotopes and the others, particularly for *A. scutatus*. The dietary literature of *A. scutatus* indicates they mostly predate on amphibians, reptiles, and mammals [37,47], having a somewhat broad dietary niche similar to *Naja*. Thus since the α-neurotoxic activity of *A. scutatus* crude venoms seems to have a specificity toward lizard mimotope, it would seem that in a similar case to *Naja*, species with a much broader generalist diet will have other equally important venom toxins (such as coagulotoxic, myotoxic, and neurotoxic PLA_2_s, etc.) that will play a key role in immobilising the broad range of different taxa prey items not particularly affected by the neurotoxins. The diet of *E. boulengeri* remains elusive within the literature, and, thus, it is difficult to make any assumptions between diet and neurotoxic selectivity toward the lizard mimotope.

**Figure 4:**
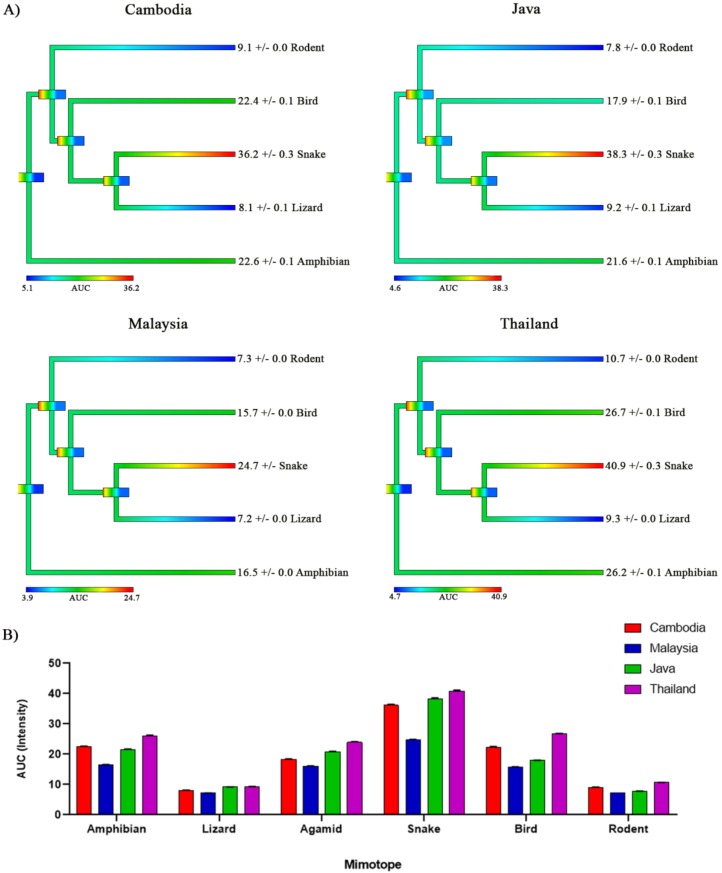
Comparisons of binding affinity of nAChR of multiple taxa to venoms from four different geographical locations (Cambodia, Java, Malaysia, and Thailand) of *Ophiophagus hannah*. (**A**) Phylogenetic tree colouring corresponds with lower AUC values (lower potency) coloured cooler, while higher AUC values (higher potency) coloured warmer. AUC values (derived from K_a_ (binding rate) curves in triplicate) ± SEM are shown next to each taxa mimotope at the tree tips. (**B**) A bar graph with the AUC of each location giving a representation of the relative target potency between each location. The SEM bars are extremely small, reflecting assay precision. All AUC values for A and B are *n* = 3.

Snakes are well documented as being the preferred prey for *O. hannah* [32,38,48,49], to the extent that the genus name means snake (‘Ophio’) eater (‘phagus’). All *O. hannah* localities studied here (Figure 4A,B) showed a high selectivity and potency for the snake mimotope (Figure 4A). Our results showing highly selective for the snake α-1 are consistent with *O. hannah* being a specialist feeder of snakes, often being difficult in captivity for their dietary specialism [50], and the venom gland transcriptome of *O. hannah* dominated with 3FTx transcripts (66.73%) [51]. In this instance it would seem that prey-specialist diets can drive the evolution of neurotoxic selectivity of the overall crude venom, which further supports our aforementioned hypothesis that α-neurotoxic prey selectivity of crude venom is likely driven by higher proportions of prey-specific 3FTxs. The data also further support the idea that prey selectivity of venom is controlled by the dietary breadth [44], with a specialised diet eliciting a more prey-specific venom effect.

Further, although all four *O. hannah* localities showed very small differences in prey targeting, large differences were evident in their binding affinity to each mimotope (Figure 4B). The notably lower potency of the Malaysian population is consistent with having a higher percentage of defensive toxins in conjunction with aposematic colouring, which is not present in the other populations [52]. These patterns suggest that differences in α-neurotoxic potency occur at different locations of *O. hannah* as a consequence of differing levels of defensive toxins. This kind of evidence may significantly inform clinical research of snakebite envenomations and antivenom administration: depending on the locale of the *O. hannah* bite, severity of neurotoxic symptoms may differ, although this difference may be masked due to the very high venom yields of these snakes. Further, these data show that this assay can also be used to test for intraspecific geographical variation in relative taxa targeting/potency and might also be useful in studies investigating ontogenetic changes in α-neurotoxic venom binding. Similarly, such tests may inform ecological assertions when field data are unavailable.

Although this assay is more taxonomically robust than other functional testing platforms (CBCM, RPND, oocyte patch clamp, etc.), it is still limited in that the binding amino acid sequences for alpha-1 cholinergic receptors (Chrna1) for mimotope design are limited in current databases, and there seem to be few species representative for certain taxa groups available. For example, the amphibian mimotope used in this study is based on the *Xenopus laevis* Chrna1 sequence, as this is the only available sequence for amphibians currently. However, the limitations of using these wide, overarching taxon models is similar to other functional venom testing assays that use the most commercially available representatives (e.g., *Gallus gallus domesticus* (CBCM), *Rattus norvegicus* (RPND), and *Mus musculus* (MPND)). Despite this current limitation, our assay has added more available taxa diversity to current neurotoxic nAChR functional testing than conventional tissue preparations, with the added element of being more ethical as no live animals had to be sacrificed for these assays. Future research will aim to sequence the Chrna1 of more individual species within certain taxa groups (if the sequences show intraspecific differences) and will be able to utilise a range of species-specific mimotopes to help further our understanding of prey-specific effects of α-neurotoxic venoms in addition to providing a novel platform upon with to explore resistance by predators or prey of neurotoxic snakes.

In summary, this innovative biolayer interferometry approach [32] enabled testing of α-neurotoxic venom selectivity across a wide range of taxon mimotopes, which facilitated the detection of prey-selective, intraspecific, and geographical differences in neurotoxic potency. Our results highlighted that elapid venoms can differ in their taxa selectivity and that 3FTxs can exhibit orthosteric preference between different taxa groups. Furthermore, we postulate that the overall prey-selective α-neurotoxicity of crude venoms is likely driven by the proportions of prey-specific toxins within the crude venom. We also show that prey-selective binding of α-neurotoxins does not always correspond directly to their diet, suggesting that other toxin functions such as coagulotoxicity, myotoxicity, or presynaptic neurotoxicity play equally vital roles in immobilising other prey types that the α-neurotoxins might not effectively target, particularly in species with broader dietary niches. Our data reinforce the hypothesis that diet breath mediates prey selectivity of venom [44]. This study also sheds light on the precise binding affinity of venoms to the orthosteric mimotopes, which can open up other potential areas of research such as ontogenetic and geographical variation in neurotoxic venom targeting. The use of this high-throughput and taxonomically diverse assay in functional venom testing is also beneficial in understanding the evolution and ecology of elapid snakes, as well as the biochemistry and pharmacology of their venom.

## 3. Materials and Methods

### 3.1. Venom Collection and Preparation

Venoms were obtained from pooled snake venom extractions from multiple individuals (captive and wild caught). Alpha-cobratoxin was purchased from Latoxan (Portes-les-Valence, France).

All venom samples were lyophilised and reconstituted in double deionised water (ddH_2_O), and then centrifuged (4 °C, 10 min at 14,000 relative centrifugal force (RCF)). The supernatant was then made into a working stock (1 mg/mL) in 50% glycerol to prevent freezing at −20 °C. The concentrations of working stocks were determined in triplicate using a NanoDrop 2000 UV-Vis Spectrophotometer (Thermo Fisher, Sydney, Australia) at an absorbance wavelength of 280 nm.

### 3.2. Mimotope Production and Preparation

Expanding upon previous research [53,54,55,56] a 13–14 amino acid mimotope of ACh orthosteric site of vertebrate α-1 nAChR subunit was developed by GenicBio Ltd. (Shanghai, China) designed upon specification, which was adapted from publicly available sequences of cholinergic receptors (Chrna1) from GenBank and UniProt.

The amino acid sequences for the α-1 orthosteric site for each taxa were obtained with the following accession codes: amphibian α-1 (uniprot F6RLA9), lizard α-1 (genbank XM_015426640), avian α-1 (uniprot E1BT92), rodent α-1 (uniprot P25108). The only exception was the α-1 sequence for the snake α-1 (*Coelognathus radiatus*), which was Sanger sequenced in a previous study [32].

The Cys–Cys of the native mimotope was replaced during peptide synthesis with Ser–Ser to avoid uncontrolled postsynthetic thiol oxidation. The Cys–Cys bond in the nAChR binding region does not participate directly in analyte-ligand binding [57,58,59], thus replacement to Ser–Ser is not expected to have any effect on the analyte-ligand complex formation. However, the presence of the Cys–Cys bridge is key in the conformation of the interaction site of whole receptors [60]. Therefore, we suggest direct comparisons of kinetics data, such as K_a_ or KD, between nAChR mimotopes and whole-receptor testing should be avoided or at least approached with caution. Mimotopes were further synthesised to a biotin linker bound to two aminohexanoic acid (Ahx) spacers, forming a 30 Å linker.

Mimotope dried stocks were solubilised in 100% dimethyl sulfoxide (DMSO) and diluted in deionised water at 1:10 dilution to create a working stock of 50 µg/mL. All stocks were stored at −80 °C until use and limited to three freeze–thaw cycles.

### 3.3. Biolayer Interferometry (BLI)

Full details of the developed assay, including all methodology and data analysis, can be found in the validated protocol [32]. In brief, the BLI assay was performed on the Octet RED 96 system (ForteBio, Fremont, CA, USA). Analyte (venom) samples were diluted 1:20 to make a final experimental concentration of 50 µg/mL per well (10 µg in each well). Mimotope aliquots were diluted 1:50 to a final concentration of 1 µg/mL per well (0.2 µg in each well). The assay running buffer was 1X DPBS with 0.1% BSA and 0.05% Tween-20. Prior to experimentation, Streptavidin biosensors were hydrated in the running buffer for 30–60 min, whilst being agitated at 2.0 revolutions per minute (RPM) on a shaker. The dissociation of analytes occurred using a standard acidic solution (glycine buffer), made up of 10 mM glycine (pH 1.5–1.7) in deionised water. Negative controls consisted of deionised water:glycerol 1:1 mix in replacement of the sample in the wells. Raw data is presented in Supplementary File 1.

### 3.4. Data Processing and Statistical Analyses

All data obtained from BLI on Octet RED 96 system (ForteBio, Fremont, CA, USA) were processed in exact accordance to the validation of this assay [32]. The association step data (in triplicate) were obtained in an excel.csv file extracted from raw outputs of the Octet Red 96 system and then imported into Prism 7.0 software (GraphPad Software Inc., La Jolla, CA, USA) where area under the curve (AUC) calculations were made and graphs produced.

Phylogenetic trees were obtained from timetree.org and then further manually recreated using Mesquite software version 3.2 (http://mesquiteproject.org/). The obtained phylogenetic trees were then further analysed in RStudio (R Core Team, 2015) for all comparative analysis using the Ape package [61]. Heat-mapping of AUC values over the phylogenetic trees was achieved using the contMap function of the R package phytools [62].

## Supplementary Materials

The following are available online at https://www.mdpi.com/2072-6651/12/3/205/s1, Supplementary File 1: Rawdata.
